# Labial Frenectomy using Laser: A Scoping Review

**DOI:** 10.1155/2023/7321735

**Published:** 2023-04-30

**Authors:** Mario Dioguardi, Andrea Ballini, Cristian Quarta, Marino Caroprese, Marta Maci, Francesca Spirito, Giorgia Apollonia Caloro, Mario Alovisi, Elisabetta Basile, Lorenzo Lo Muzio

**Affiliations:** ^1^Department of Clinical and Experimental Medicine, University of Foggia, Via Rovelli 50, Foggia 71122, Italy; ^2^Department of Precision Medicine, University of Campania “Luigi Vanvitelli”, Naples 80138, Italy; ^3^Unità Operativa Nefrologia e Dialisi, Presidio Ospedaliero Scorrano, ASL (Azienda Sanitaria Locale) Lecce, Via Giuseppina Delli Ponti, Scorrano 73020, Italy; ^4^Department of Surgical Sciences, Dental School, University of Turin, Turin 10127, Italy

## Abstract

Labial frenectomy is a surgical technique, that aims to remove the frenulum with its attachment to the underlying bone. Frenectomy, is indicated if the frenulum attachment causes midline diastema, gingival recession, hindrance in maintaining oral hygiene, or if it interferes with lip movements and for prosthetic needs. A labial frenectomy can be performed either by the routine scalpel technique, electrocautery, and most recently medical lasers. The aim of this study was to evaluate, whether the laser technique is more effective than the conventional surgical technique, and whether there are differences between the different types of lasers. The scoping review was conducted and prepared on the basis of the indications of the PRISMA guidelines (PRISMA Extension for Scoping Reviews, PRISMA-ScR) of PRISMA checklist, and nine papers were considered admissible to the qualitative analysis for the following outcomes: bleeding during intervention, use of sutures, duration of the intervention, and use of analgesic drugs in the days following the intervention. This review suggests that laser-performed labial frenectomy is faster and offers better intra- and postoperative management; however, due to the limited number of available papers, the final results of the present review are not absolute.

## 1. Introduction

The labial frenulum is a thin fold of the mucous membrane composed of connective tissue and muscle fibers that attaches the lips to the alveolar mucosa or gingiva by periosteal insertion [1]; in many cases, the frenulum may compromise dental positioning and restrict labial movement because of its attachment localization, thus affecting the stability of prosthetics manufacturing, phonation, and esthetics of the patient [2].

There is a classification for the different types of the upper and lower frenulum attachment, which considers the extension of the attachment and supports one to indicate the cases suitable for the prophylactic frenulectomy [3]:

- Mucosal attachment, which is the most common in both jaws (42%)
- Gingival attachment, which is the second most frequent type (34%)
- Papillary attachment (20%) and papilla penetrating attachment (4%), which are the least common [4]

The papillary attachment and papilla penetrating attachment types regularly caused the pull syndrome, a detaching movement of the marginal gingiva transferred from the lip by the frenulum, that also occurs in many cases of gingival types, being rather rare in the mucosal type; furthermore, the papillary type in both jaws, the gingival type and the papilla penetrating type in lower jaw, seemed to be related with the pathologically modified midline interdental papillae, in the highest percentage of cases [3].

Labial frenectomy is a surgical technique, that aims to remove the frenulum with its attachment to the underlying bone [1]. Frenectomy, is indicated if the frenulum attachment causes midline diastema, gingival recession, hindrance in maintaining oral hygiene, or if it interferes with lip movements and for prosthetic needs [5–7].

In oral surgery, additionally to labial frenectomy, lingual frenectomy should also be taken into consideration as a possible solution to complications such as ankyloglossia, a very common issue that affects both children and adults. In this case, when the function is impaired, the release of the lingual frenulum must be considered, and the use of diode laser has proved to be an effective alternative for the treatment of ankyloglossia, as demonstrated by an observational study conducted on 32 consecutive cases, evaluating the degree of recurrence of ankyloglossia after diode laser lingual frenectomy [8].

Labial frenectomy can be performed either by the routine scalpel technique, electrocautery, and most recently medical lasers [5], including the Nd:YAG, the diode, the CO_2_, the Er:YAG, and the Er, Cr:YSGG laser [9].

The excision of the frenum by using a scalpel, which is the surgical technique used routinely, carries the routine risks of surgery like bleeding and patient compliance [5], while medical lasers provide excellent hemostasis [10] and cause a lesser degree of injury to the surrounding tissue and limited scarring, with reduced pain and edema, and consequently, greater postoperative comfort, as they possess the ability to selectively and precisely interact with injured tissue [6, 11, 12].

Laser treatments are employed in oral surgery for some procedures, such as gingivectomies, frenectomies, operculum removal, and benign lesions biopsies [9].

The aim of this study was to evaluate whether the laser technique is more effective than the conventional surgical technique and whether there are differences between the various types of lasers for the following variables: bleeding, mean surgical time, need for suturing, and taking analgesic drugs.

## 2. Materials and Methods

The following scoping review was conducted on the basis of the indications of the PRISMA protocol for scoping reviews:

The study was developed on PICO question (Population, Intervention, Control, and Outcome): population (patients treated for labial frenulectomy), intervention (laser technique was used to perform labial frenectomy), comparison (was compared between the use of different types of lasers and the conventional surgical technique), and outcome (it was evaluated whether the laser technique was more effective than the conventional surgical technique, and whether there were differences between the different types of lasers, for the following variables: bleeding, time taken to perform the operation, need for sutures, use of analgesic drugs).

The wording of the PICO question was as follows: which technique generated less bleeding during surgery? Where were the sutures required at the end of the treatment? Were there differences in the duration of the different treatments? Had the patients used analgesic drugs in the days following the surgical intervention? After a first phase of records identified in the PubMed and Scopus database and of papers selected by bibliographic references of other sources, the selection of potential admissible papers was evaluated qualitatively, in order to identify differences between the different types of intervention.

### 2.1. Eligibility Criteria

The studies taken into consideration were randomized clinical studies, comparative studies, retrospective studies, and case reports, that have assessed the use of the laser in frenulectomy interventions, published with abstract in English.

The potentially admissible papers were finally subjected to a full-text analysis, to verify its use for a qualitative analysis and quantitative analysis.

The following criteria have been applied in full-text analysis:

Inclusion: all those studies that have assessed the use of the laser in labial frenectomy interventions.

Exclusion: studies that considered patients with systemic diseases, such as hemorrhagic disorders, constant use of drugs, metabolic diseases, and studies published in language other than English.

### 2.2. Research Strategy

Studies have been identified through bibliographic research on the PubMed and Scopus database.

The research was conducted between June 1, 2022, and July 4, 2022; the following research terms were used:

“Labial Frenectomy” and “Laser” and “Frenectomy”

The research of the records and their selection was conducted by two authors (MD) and (AB), supported from a 3rd author (CQ), with the role of resolving doubtful and conflicting situations. In detail, the following search was conducted on PubMed with the following terms, as depicts in Table 1.

In addition, a gray literature review was conducted by consulting Google Scholar, ScienceDirect, and OpenGrey.

As a complement to this research, manual research of the papers included in the bibliographic references of other sources was performed.

### 2.3. Methodology Screening

The research of the records and their selection was conducted by two authors (MD) and (AB) with a 3rd author (CQ) with the role of resolving doubtful and conflicting situations. Initially, the papers were selected by analyzing title and abstract according to the research previously described, and in doubtful cases, the text was also analyzed to eliminate records not relating to the topics of the review.

## 3. Results

**Figure 1:**
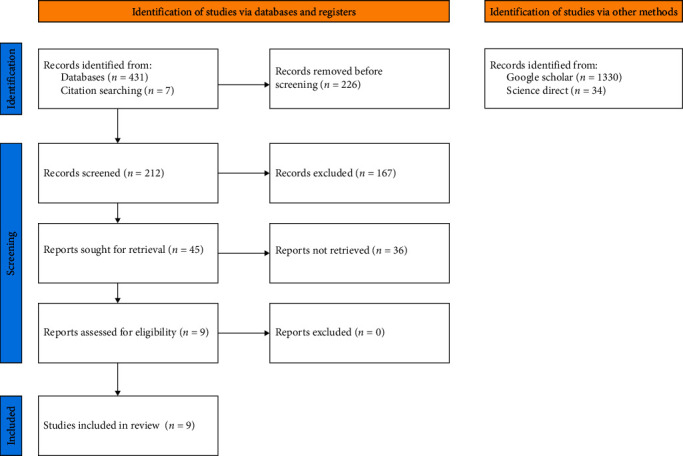
Flowchart of study identification.

After screening the database Scopus and PubMed (Labial Frenectomy: 109 Records PubMed, 123 Records Scopus; Laser and Frenectomy: 96 Records PubMed, 110 Records Scopus), and manually searching for papers included in the references from other sources, 212 studies were identified. After the evaluation of the papers by title and abstract, 45 were selected for the evaluation of the full text, and of these, only nine were selected for qualitative and quantitative analysis.  Figure 1 shows the flowchart of the different phases of the search strategy.

The selected studies included in this scoping review are the follows: Olivi et al. [13]; Olivi et al. [7]; Sarmadi et al. [14]; Júnior et al. [15]; Pié-Sánchez et al. [16]; Gargari et al. [17]; Do Hoang et al. [18]; Patel et al. [19]; and Akpınar et al. [20].

### 3.1. Characteristics of the Included Studies

The characteristics of the nine selected studies are listed in Table 2.

The data were collected for reference, study design, number of patients, age, groups, bleeding during surgery, sutures, mean surgical time, and analgesic consumption.

Totally, three studies were retrospective studies, four were randomized controlled trial, one was a comparative study and one was a case report.

The study that analyzed the largest number of patients was that of Olivi et al. [7] with 143 patients, and generally there were a similar number of male and female patients, while in one study the sex of the patients was not determined [13].

In five studies, age was expressed in range: Olivi et al. [13], Olivi et al. [7], Sarmadi et al. [14], Júnior et al. [15], and Do Hoang et al. [18], while in three studies it was expressed as mean age: Pié-Sánchez et al. [16], Patel et al. [19], and Akpınar et al. [20].

Among lasers, the diode laser was studied in three studies: Gargari et al. [17], Do Hoang et al. [18], and Patel et al. [19], Er:YAG laser in two studies [13, 14], Nd:YAG laser in two studies [15, 20], Er, Cr:YSGG laser in two studies [7, 16], and the CO_2_ laser in only one study [16]; the comparison with the traditional surgical technique was performed in four studies: Sarmadi et al. [14], Júnior et al. [15], Patel et al. [19], and Akpınar et al. [20].

### 3.2. Bleeding during Surgery, Sutures, Mean Surgical Time, and Analgesic Consumption

Table 2 also shows the data relating to bleeding during surgery, sutures, mean surgical time, and analgesic consumption.

Only two studies reported no bleeding data [7, 20]; one study reported the average amount of blood expressed in milligrams [14], in another study bleeding was expressed as an average value on a scale ranging from a minimum of 1, indicating no bleeding, to a maximum of 4, indicating profuse bleeding [19]. Intraoperative bleeding was present in all scalpel surgeries, except in one in which the value was not reported [20], and reported higher values than the laser group with which it was compared in each single study. The absence of bleeding occurred in the CO_2_ laser group in the study of Pié-Sánchez et al. [16] and in the laser diode group in the study of Gargari et al. [17], while the bleeding occurred only in one patient in the Nd:YAG laser group, in the study of Júnior et al. [15], and only in six patients in the laser diode group in the study of Do Hoang et al. [18].

It was necessary to place the sutures in all the scalpel surgeries, while between the laser groups, it was necessary to suture only in two patients of the Er:YAG laser group, as reported in the study of Sarmadi et al. [14]; moreover, only in one study the suture data were not reported [16].

Surgical time was not reported in four studies (Table 2), and was found to be greater in the scalpel surgery groups than in each comparing group.

Analgesic consumption was not reported in one study [20], while in three studies, no patient undergo to pain drugs: Olivi et al. [13], Olivi et al. [7], and Gargari et al. [17].

## 4. Discussion

The present study, is a systematic review of the literature concerning labial frenectomy performed with laser or scalpel evaluating its efficacy through different outcomes such as bleeding, execution time, type of suture, and analgesics. The review at the end of the paper selection phase included nine studies with a total of 433 patients participating in the different groups.

The type of attachment, and the extent of the labial frenulum, allow us to indicate suitable cases for prophylactic frenectomy, as they involve the presence of functional problems and seemed to be related with the pathologically modified midline interdental papillae [3]. Scalpel frenectomy, the most commonly used method, is associated with postoperative pain and discomfort, and this procedure also requires sutures which can lead to greater patient discomfort and complications; laser surgery is a great alternative to scalpel for such surgeries because laser treatment does not require sutures in most cases, reduced surgical time, less postoperative pain, and discomfort leading to greater patient acceptance [21].

The use of lasers in dental surgery is already established as an effective option to minimize the pain and discomfort of the patient during and after surgical intervention [22, 23]. The data from these reviews confirm this trend.

The variables measured included bleeding during surgery, mean surgical time, need for suturing, and use of analgesic drugs. The use of a high-intensity laser presents significant advantages in the oral surgical procedures, including less bleeding due to the power of hemostasis [24] and significant reduction in the need for sutures [10].

In the studies included in this review, when the laser technique was compared with traditional surgery, bleeding during surgery was always less in terms of both mean amount [14, 19] and prevalence among patients [15]; this can be attributed to high-temperature coagulation of soft tissue proteins resulting in reduced bleeding at the edges of the ablated tissue and narrowing of blood vessel walls due to high temperatures thus causing photothermal coagulation [25].

An advantage of using laser surgery is the possibility of avoiding or reducing the consumption of analgesic drugs and thus reducing postoperative pain [24], because the laser causes minimal collateral damage and also brings about sealing of lymphatics, also forming a fibrin clot on the surgical site that protects it from external irritation [16]; increased numbers of analgesics have been found due to postoperative pain when using the conventional scalpel technique, which can be caused by involvement of a large surgical site causing more blood loss, a larger wound, and the need for suturing; sutures can cause postoperative discomfort due to the accumulation of food and plaque [26, 27]. In the studies in which only the use of lasers was evaluated, no use of analgesics was reported [7, 13, 17] or they were in any case taken by a small number of patients [18]. Only one study reports a higher prevalence of taking analgesic drugs in the traditional surgical technique group (scalpel) [19, 28], while in the other studies there were no obvious differences [14, 15]. In the study in which two laser techniques were evaluated [16], i.e. CO_2_ and Er, Cr:YSGG laser, there were no differences in the intake of analgesic drugs, while intraoperative bleeding occurred only in the Er, Cr:YSGG laser group. Many authors agree that laser treatment has led to a more comfortable postoperative period with less pain and edema [2, 29, 30].

The laser can allow you to make incisions in the tissue without the need for sutures as they cause little or no bleeding, which makes it a quick and easy outpatient procedure [31]. Only in one study it was necessary to suture the surgical wound in only two patients [14], while in all the other studies the laser technique did not require any suturing (Table 2). In the studies included in this review that evaluated surgical time, this was lower in laser treatment than in traditional surgery [14, 15, 20], confirming what has already been reported in the literature [31–33], and according to the evidence-based dentistry [34].

The limitations of the present scoping review are the low number of included studies and the high-heterogeneity of the data which makes a quantitative evaluation of the data by meta-analysis inconsistent. However, higher cost and need for operator skill are the associated limitations.

## 5. Conclusion

This scoping review suggests that laser-performed labial frenectomy is faster and offers better intra- and postoperative management. Lasers have the advantage of better patient acceptance due to reduced pain perception and postoperative discomfort. Furthermore, reduced intraoperative bleeding is encountered compared to scalpel. However, due to the low number of papers included in the review, it cannot yet be said that laser surgery is better than traditional surgery in labial frenectomy and if there are important differences between the various types of laser; furthermore, there is a lack of studies that evaluate the various surgical techniques taking into consideration the type of insertion of the frenulum, which is an important parameter for evaluating the decision to perform frenectomy; therefore these results should be viewed with caution [35].

## Figures and Tables

**Table 1:** In detail the research conducted on PubMed on July 4, 2022.

Search: (Laser AND Frenectomy) OR Labial Frenectomy Sort by: Most Recent
((“laser s”[All Fields] OR “lasers”[MeSH Terms] OR “lasers”[All Fields] OR “laser”[All Fields] OR “lasered”[All Fields] OR “lasering”[All Fields]) AND (“fraenectomy”[All Fields] OR “frenectomies”[All Fields] OR “frenectomy”[All Fields])) OR ((“labially”[All Fields] OR “lip”[MeSH Terms] OR “lip”[All Fields] OR “labial”[All Fields] OR “labials”[All Fields]) AND (“fraenectomy”[All Fields] OR “frenectomies”[All Fields] OR “frenectomy”[All Fields])). Translations:Laser: “laser's”[All Fields] OR “lasers”[MeSH Terms] OR “lasers”[All Fields] OR “laser”[All Fields] OR “lasered”[All Fields] OR “lasering”[All Fields] Frenectomy: “fraenectomy”[All Fields] OR “frenectomies”[All Fields] OR “frenectomy”[All Fields]. Labial: “labially”[All Fields] OR “lip”[MeSH Terms] OR “lip”[All Fields] OR “labial”[All Fields] OR “labials”[All Fields]. Frenectomy: “fraenectomy”[All Fields] OR “frenectomies”[All Fields] OR “frenectomy”[All Fields].

**Table 2:** Characteristic of the included studies with the main data extracted from the papers, No. (number), n.r. (data not present), M (male), F (female), min (minutes), s (seconds), and mg (milligrams).

No.	Reference	Study design	Groups	No. of patients (M/F)	Age (years)	Bleeding during surgery	Sutures	Mean surgical time	Analgesic consumption
1	Olivi et al. [13]	Retrospective study	Er:YAG laser	20 (n.r.)	8–10	Yes	No	n.r.	No

2	Sarmadi et al. [14]	Randomized controlled trial	Scalpel	40 (10/30)	8–13	1,080 mg	Yes	10 min 35 s	Yes (11) No (9)
			Er:YAG laser			332 mg	Only in two patients	6 min 52 s	Yes (9) No (11)

3	Júnior et al. [15]	Comparative study	Scalpel	40 (16/24)	8–51	Yes	Yes	10 min 12 s	Yes (16) No (6)
			Nd:YAG laser			Only in one patient	No	7 min 42 s	Yes (13) No (5)

4	Olivi et al. [7]	Retrospective study	Er,Cr:YSGG laser	143 (73/70)	7–11	n.r.	No	7–10 min	No

5	Pié-Sánchez et al. [16]	Randomized controlled trial	CO_2_ laser	50 (22/28)	11.3	No	n.r.	49.50 s	Yes (1) No (24)
			Er,Cr:YSGG laser			Yes	n.r.	162.56 s	Yes (1) No (24)

6	Gargari et al. [17]	Case report	Diode laser	1 (0/1)	32	No	No	n.r.	No

7	Do Hoang et al. [18]	Retrospective study	Diode laser	30 (20/10)	7–14	Yes (6) No (24)	No	n.r.	Yes (5) No (25)

8	Patel et al. [19]	Randomized controlled trial	Scalpel	20 (8/12)	32.4 ± 7.75	2.37 ± 0.51	Yes	n.r.	4.25 ± 0.7
			Diode laser			0.25 ± 0.46	No	n.r.	1.87 ± 0.83

9	Akpınar et al. [20]	Randomized controlled trial	Scalpel	89 (38/51)	28.75 ± 11.32	n.r.	Yes	9.93 ± 3.32	n.r.
			Nd:YAG laser		29.75 ± 11.58	n.r.	No	8.84 ± 3.11	n.r.

No. (number), n.r. (data not present), M (male), F (female), min (minutes), s (seconds), and mg (milligrams).

## Data Availability

The lierature data supporting this scoping review are from previously reported studies and datasets, which have been cited. The processed data are available within the text.
